# Apparent treatment-resistant hypertension associated lifetime cardiovascular risk in a longitudinal national registry

**DOI:** 10.1093/eurjpc/zwad066

**Published:** 2023-03-02

**Authors:** Joseph E Ebinger, Anni Kauko, FinnGen, Natalie A Bello, Susan Cheng, Teemu Niiranen

**Affiliations:** Department of Cardiology, Smidt Heart Institute, Cedars-Sinai Medical Center, 127 S San Vicente Blvd a3600, Los Angeles, CA, USA; Department of Internal Medicine, University of Turku, Kiinamyllynkatu 4-8, 20521 Turku, Finland; Department of Cardiology, Smidt Heart Institute, Cedars-Sinai Medical Center, 127 S San Vicente Blvd a3600, Los Angeles, CA, USA; Department of Cardiology, Smidt Heart Institute, Cedars-Sinai Medical Center, 127 S San Vicente Blvd a3600, Los Angeles, CA, USA; Department of Internal Medicine, University of Turku, Kiinamyllynkatu 4-8, 20521 Turku, Finland; Division of Medicine, Turku University Hospital, Kiinamyllynkatu 4-8, 20521 Turku, Finland; Department of Public Health Solutions, Finnish Institute for Health and Welfare, Mustionkatu 10b, 20750 Turku, Finland

**Keywords:** Hypertension, Resistant hypertension, Renal failure, Stroke, Heart failure

## Abstract

**Aims:**

Apparent treatment-resistant hypertension (aRH), wherein blood pressure elevation requires treatment with multiple medications, is associated with adverse cardiovascular events over the short-term. We sought to evaluate the degree of excess risk associated with aRH across the lifespan.

**Methods and results:**

We identified all individuals with hypertension who were prescribed at least one anti-hypertensive medication from the FinnGen Study, a cohort of randomly selected individuals across Finland. We then identified the maximum number of concurrently prescribed anti-hypertensive medication classes prior to age 55 and classified those co-prescribed ≥4 anti-hypertensive medication classes as aRH. Using multivariable adjusted Cox proportional hazards models, we assessed the association of aRH well as the number of co-prescribed anti-hypertensive classes with cardiorenal outcomes across the lifespan. Among 48 721 hypertensive individuals, 5715 (11.7%) met the aRH criteria. Compared to those prescribed only one anti-hypertensive medication class, the lifetime risk of renal failure increased with the addition of each additional medication class, beginning with the second, while the risk of heart failure and ischaemic stroke increased after addition of the third drug class. Similarly, those with aRH suffered increased risk of renal failure (hazard ratio 2.30, 95% CI 2.00–2.65), intracranial haemorrhage (1.50, 1.08–2.05), heart failure (1.40, 1.24–1.63) cardiac death (1.79, 1.45–2.21), and all-cause death (1.76, 1.52–2.04).

**Conclusion:**

Among individuals with hypertension, aRH that develops prior to mid-life is associated with substantially elevated cardiorenal disease risk across the lifespan.

## Graphical Abstract

**Graphical Abstract:**
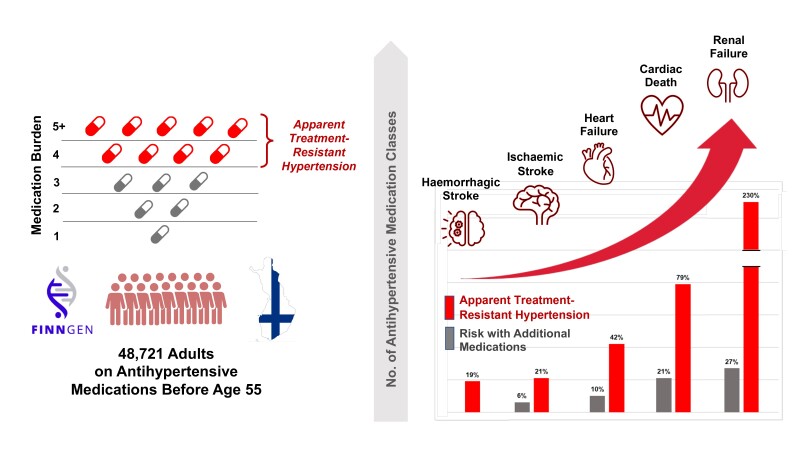
The risk of cardiorenal events increases with the addition of greater number of antihypertensive medications and is highest among patients with apparent treatment-resistant hypertension.

## Introduction

Awareness of resistant hypertension, defined as blood pressure above goal despite the use of at least three antihypertensive medications or achieving target blood pressure with four or more medications, has increased in recent years, due in part to reductions in goal blood pressure targets for large portions of the population. Numerous non-biological factors, such as medication non-adherence, white coat effect, and submaximal drug dosing, should be excluded prior to diagnosing resistant hypertension. When such conditions cannot be excluded, the term apparent treatment-resistant hypertension (aRH) is utilized, a criterion met by an estimated 12–15% of treated antihypertensive patients.^1–7^

Limited short-term analyses, over the course of less than 5 years, demonstrate that patients with resistant hypertension are nearly 50% more likely to suffer myocardial infarction, stroke, heart failure, kidney disease, or death than individuals with essential hypertension.^8^ Importantly, hypertension attributed cardiovascular risk is experienced over the entire lifespan, not only the short time frames examined in prior studies. As such, it remains unknown if, and to what degree, elevated cardiovascular risk attributed to resistant hypertension persists or if the cumulative effect of essential hypertension closes this gap overtime. As such, we sought to address this gap by evaluating the development of adverse cardiovascular outcomes across the lifespan between hypertensive patients with and without aRH.

## Methods

The Finnish Biobank data can be accessed through the Fingenious^®^ services (https://site.fingenious.fi/en/) managed by the FINBB.

### Study sample

Our study sample was drawn from the FinnGen Data Freeze 8, which consists of randomly selected participants from Finnish cohort studies and patients from Finland’s national hospital biobanks.^9^ Briefly, FinnGen represents a public–private partnership that longitudinally collects and manages anonymous nationwide health information from across Finland including diagnoses, medications, clinical events, health registry, and genomic data. From this sample, we identified patients with a diagnosis of hypertension and prescription of at least one antihypertensive medication prior to 55 years of age. Participants with a history of any of the outcomes of interest prior to age 55 were excluded. These outcomes include coronary heart disease (CHD), ischaemic stroke, haemorrhagic stroke, heart failure, renal failure, and a cardiovascular disease (CVD) composite outcome consisting of CHD and ischaemic and haemorrhagic stroke. We also assessed for cardiovascular death and all-cause mortality. Outcomes were identified based on International Classification of Diseases (ICD) codes (see Supplementary material online, *Table S1*). All participants provided written informed consent. This study protocol was approved by the coordinating ethical committee of the Hospital District of Helsinki and Uusimaa, as described in the Supplementary material online, *Methods*.

### Exposure definition

Clinical diagnoses were identified from ICD codes from the nationwide hospital discharge, causes-of-death, drug reimbursement, and drug purchase registers and linked by personal nationwide identification codes. These ICD code–based diagnoses are made by the attending physician and are listed in the Supplementary material online, *Table S1*. The accuracy of these codes is robust and has been described in detail previously.^10^ A priori selected covariates included sex (inferred from genetic data), diabetes, obesity, and hypercholesterolaemia. Covariate data for the main analysis were complete. Disease variables were defined based on the presence or absence of events in appropriate registries, and the FinnGen core analysis excludes individuals with ambiguous sex.

Antihypertensive medication prescription was defined by the Anatomical Therapeutic Chemical (ATC) codes via the drug purchase register (available from 1992 to 2019). Antihypertensive medications were divided into six classes: diuretics, angiotensin-converting enzyme (ACE) inhibitors, angiotensin II receptor blockers, calcium channel blockers, beta blockers, and other medications (see Supplementary material online, *Table S2*). Loop diuretics were excluded from the analysis to avoid confounding from undocumented chronic kidney disease or heart failure, for which this class of medications is typically used.^11^ For each individual, we determined the maximum number of different antihypertensive classes prescribed during any 3-month period before age 55, corresponding with the maximum 3-month supply that can be reimbursed at one transaction by the Finnish pharmacies. To guarantee that exposure variables were derived from the time period prior to outcome events, we included only purchases prior to 55 years age. We then divided participants into discrete hypertensive subgroups of interest including mild hypertension (one drug class) and apparent treatment resistant hypertension (four or more drug classes). We used a conservative definition of apparent treatment resistant hypertension, requiring prescription at least four drug classes, as we were unable to determine blood pressure control to meet lower prescription thresholds.^12^ Further, as has been done by others, we excluded the requirement for use of a diuretic, as intolerance or electrolyte abnormalities may preclude their use.^8^

### Statistical analyses

We used Cox proportional hazards model to assess the association between the maximal number antihypertensive medication classes and outcomes. Maximal prescription of one antihypertensive drug class within 3 months was used as the reference category. The follow-up spanned from 1992 to 2019, and participants were censored at the end of follow-up, death, or at the time of the outcome event. We used age as timescale and sex, diabetes, obesity, and hypercholesterolaemia as covariates in all models. Covariate data were available from 1969 to 2019. We validated assumptions for the Cox model by visual inspection of graphs: proportional hazards assumption by log-minus-log plots, goodness of fit by Cox–Snell residuals, influential observations by DFBETAS, and linearity by martingales. We used R v.4.1.2 for all analyses.

### Sensitivity analyses

We performed sensitivity analyses as follows: (i) dividing diuretics to thiazides/thiazide-like diuretics and potassium-sparing diuretics, (ii) including Fine and Gray sub-distribution models to account for death, and (iii) including educational level, smoking, and alcohol abuse as a covariate in those with available data. Educational level (25% missing) and smoking (42% missing) were excluded from the main analysis due to the high missingness.

## Results

A total of 342 499 Finnish individuals were identified in the FinnGen Data Freeze 8, of whom 48 721 (14.2%) had diagnosis of hypertension, prescription of at least 1 antihypertensive medication, and no history of any of the outcomes prior to the age of 55. Of this cohort, the average age was 48.1 ± 6.7 years, with 25 315 (52.0%) women, 10 616 (21.8%) suffering from diabetes, 4160 (8.5%) from obesity, and 16 425 (33.7%) from hypercholesterolaemia. The most frequent antihypertensive medication class during a 3-month period was beta blockers (*n* = 36,975, 33.2%; Supplementary material online, *Table S3*). The prevalence of diabetes, obesity, and hypercholesterolaemia, as well as most clinical outcomes, increased with the number of medication classes prescribed. As the number of medication classes prescribed increased, the proportion of individuals with at least a bachelor’s degree declined, while the proportion who smoked or with documented alcohol abuse increased (see Supplementary material online, *Table S4*). A total of 5715 (11.7%) individuals met the criteria for apparent treatment resistant hypertension, with prescription of four or more antihypertensive medication classes (*Table 1*). Among those with aRH, the average age at the first time of maximal number of co-prescribed medications was 48.1 ± 6.4, and 41.4% were female; the most common comorbidity was hypercholesterolaemia (54.2%).

**Table 1:** Characteristics of hypertensive individuals by maximal number of co-prescribed antihypertensive medication classes and apparent treatment-resistant hypertension status. Mild hypertension includes individuals with a diagnosis of hypertension receiving maximally one antihypertensive medication class, while apparent treatment-resistant hypertension requires four or more medication classes. Differences in prevalence between individuals with mild hypertension and resistant hypertension were tested by the chi-square test

	1 (mild hypertension)	2	3	4	5+	Apparent treatment-resistant hypertension (four or more medication classes)	*P*-value
**Characteristics**							
*n*	15 045	16 339	11 622	4893	822	5715	—
Female	8140 (54.1%)	8844 (54.1%)	5964 (51.3%)	2094 (42.8%)	273 (33.2%)	2367 (41.4%)	<0.001
Diabetes mellitus	2052 (13.6%)	3101 (19.0%)	3169 (27.3%)	1836 (37.5%)	458 (55.7%)	2294 (40.1%)	<0.001
Obesity	690 (4.6%)	1232 (7.5%)	1332 (11.5%)	755 (15.4%)	151 (18.4%)	906 (15.9%)	<0.001
Hypercholesterolaemia	3295 (21.9%)	5136 (31.4%)	4898 (42.1%)	2559 (52.3%)	537 (65.3%)	3096 (54.2%)	<0.001
**Outcomes**							
Cardiovascular composite^a^	2892 (23.2%)	2288 (18.6%)	1379 (17.2%)	516 (16.6%)	91 (20.1%)	607 (17.0%)	<0.001
Coronary heart disease	2119 (16.7%)	1674 (13.3%)	1017 (12.2%)	410 (12.7%)	74 (15.4%)	484 (13.0%)	<0.001
Heart failure	1277 (9.7%)	1066 (8.1%)	648 (7.5%)	278 (8.3%)	32 (6.7%)	310 (8.1%)	0.002
Ischaemic stroke	1101 (9.0%)	885 (7.1%)	549 (6.5%)	201 (6.1%)	30 (6.0%)	231 (6.1%)	<0.001
Haemorrhagic stroke	234 (2.0%)	190 (1.6%)	104 (1.3%)	46 (1.5%)	8 (1.7%)	54 (1.5%)	0.044
Renal failure	838 (6.3%)	826 (6.2%)	646 (7.3%)	318 (9.2%)	49 (10.0%)	367 (9.3%)	<0.001
Death	1019 (7.7%)	881 (6.5%)	580 (6.4%)	244 (6.7%)	39 (7.0%)	283 (6.8%)	0.053
Cardiac death	484 (3.6%)	432 (3.2%)	324 (3.6%)	127 (3.5%)	17 (3.1%)	144 (3.4%)	0.56

Cardiovascular outcomes represent a composite of coronary heart disease, ischaemic stroke, and haemorrhagic stroke.

### Outcomes

The most common individual cardiorenal outcome across the entire population was CHD (10.9%), followed by heart failure (6.8%), and ischaemic stroke (5.7%). The composite of CHD, ischaemic stroke, and haemorrhagic stroke occurred in 7166 (14.7%) individuals. A total of 2763 (5.7%) deaths occurred during follow-up, with 1388 (2.8%) suffering cardiac death (*Table 2*). While females represented a majority of the cohort population (52.0%), they comprised less than half of events across all measured outcomes, with the highest proportion of women appreciated for haemorrhagic stroke (46.7%). Smoking was the most common comorbid condition among each outcome. Frequency of each outcome decreased with increasing levels of educational attainment.

**Table 2:** Frequency of cardiorenal outcomes and mortality by sex, clinical comorbidities, and educational attainment

	Overall	Cardiovascular composite	Coronary heart disease	Heart failure	Ischaemic stroke	Haemorrhagic stroke	Renal failure	Death	Cardiac death
*n*	48 721	7166 (14.7%)	5294 (10.9%)	3301 (6.8%)	2766 (5.7%)	582 (1.2%)	2677 (5.5%)	2763 (5.7%)	1388 (2.8%)
Female	25 315 (52.0%)	2984 (41.6%)	2003 (37.8%)	1288 (39.0%)	1211(43.8%)	272 (46.7%)	981 (36.6%)	971 (35.1%)	394 (28.4%)
Diabetes mellitus	10 616 (21.8%)	1399 (19.5%)	1130 (21.3%)	721 (21.8%)	547 (19.8%)	83 (14.3%)	849 (31.7%)	726 (26.3%)	424 (30.5%)
Obesity	4160 (8.5%)	274 (3.8%)	201 (3.8%)	159 (4.8%)	111 (4.0%)	18 (3.1%)	170 (6.4%)	149 (5.4%)	89 (6.4%)
Hypercholesterolaemia	16 425 (33.7%)	1908 (26.6%)	1544 (29.2%)	893 (27.1%)	756 (27.3%)	141 (24.2%)	920 (34.4%)	783 (28.3%)	451 (32.5%)
Alcohol abuse	5094 (10.5%)	861 (12.0%)	618 (11.7%)	519 (15.7%)	387 (14.0%)	82 (14.1%)	485 (18.1%)	645 (23.3%)	342 (24.6%)
Smoking^a^	14 893 (52.7%)	2486 (55.4%)	1895 (58.3%)	1289 (60.6%)	1008 (53.8%)	208 (55.9%)	961 (58.2%)	1334 (66.9%)	715 (68.8%)
Educational attainment^b^									
Secondary	19 610 (53.8%)	2569 (56.4%)	1921 (57.4%)	1178 (59.1%)	986 (56.8%)	196 (55.5%)	970 (56.2%)	981 (62.6%)	463 (64.1%)
Post-secondary	8721 (23.9%)	1057 (23.2%)	782 (23.4%)	463 (23.2%)	392 (22.6%)	76 (21.5%)	380 (22.0%)	323 (20.6%)	138 (19.1%)
Higher	8122 (22.3%)	929 (20.4%)	641 (19.2%)	351 (17.6%)	359 (20.7%)	81 (22.9%)	375 (21.7%)	264 (16.8%)	121 (16.8%)

Proportions based on *n* = 28 286 individuals for whom smoking data was available.

Proportions based on *n* = 36 453 individuals for whom educational attainment data was available.

### Risk of outcomes by number of antihypertensive medication classes and apparent treatment-resistant hypertension status

**Figure 1:**
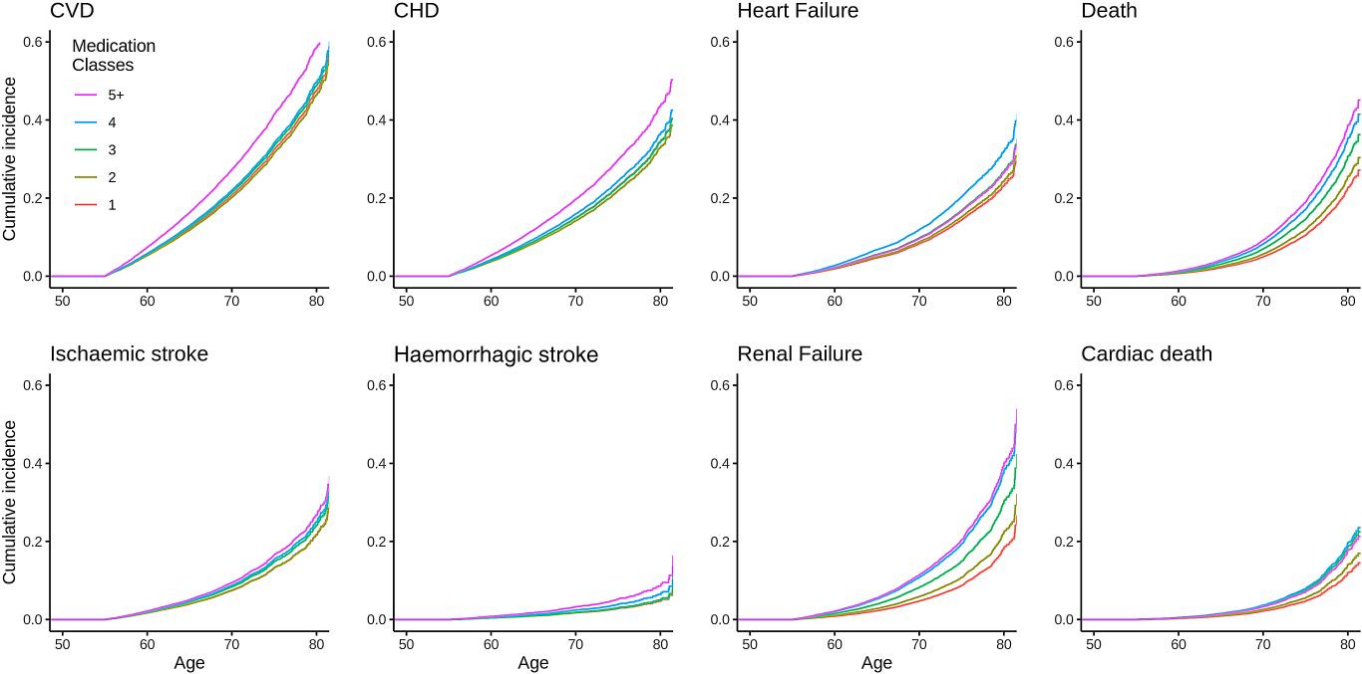
Cox proportional hazard models for adverse cardiovascular events over the lifespan, by number of antihypertensive medication classes. Hard cardiovascular outcomes (CVD) represent a composite of coronary heart disease, ischaemic stroke, haemorrhagic stroke, death, and cardiac death. CVD, hard cardiovascular outcomes; CHD, coronary heart disease. Follow-up and risk of events begins at age 55 year for all participants.

The risk of experiencing each of the pre-specified outcomes of interest generally increased as the number of antihypertensive medications prescribed increased. Compared to hypertensive patients receiving a maximum of one antihypertensive class, the lifetime risk of renal failure and death increased with the addition of each successive medication class, beginning with the second, while the risk of heart failure and ischaemic stroke started to increase after the addition of the third drug class. Significant elevations in risk of CHD and CVD were not appreciated until the addition of the 5th medication class. There was a trend towards increased risk of haemorrhagic stroke with increasing medication classes, but this did not reach statistical significance (*Table 3*, *Figure 1*, and Supplementary material online, *Figure S1*). Overall, a trend of increased risk for heart failure (hazard ratio 1.1, 95% confidence interval 1.06–1.14), renal failure (1.32, 1.27–1.37), ischaemic stroke (1.06, 1.02–1.10), CVD (1.03, 1.00–1.05), death (1.19, 1.14–1.24), and cardiac death (1.21, 1.15–1.28) occurred with the addition of each new medication class.

**Table 3:** Cardiovascular, renal, and mortality outcomes of hypertension by number of medication classes. Cox models were adjusted for age, sex, obesity, diabetes, and hypercholesterolaemia. Apparent treatment-resistant hypertension includes individuals maximally on at four or more medication classes

Medication classes	Cardiovascular outcomes^a^	Coronary heart disease	Heart failure	Ischaemic stroke	Haemorrhagic stroke	Renal failure	Death	Cardiac death
** **2	0.97 (0.92–1.02)	0.95 (0.89–1.01)	1.06 (0.97–1.15)	1.00 (0.91–1.09)	1.02 (0.84–1.24)	1.25 (1.13–1.38)	1.14 (1.04–1.25)	1.19 (1.04–1.35)
** **3	1.04 (0.97–1.11)	1.00 (0.92–1.08)	1.18 (1.07–1.30)	1.13 (1.01–1.25)	1.06 (0.84–1.34)	1.77 (1.59–1.97)	1.42 (1.28–1.58)	1.62 (1.40–1.88)
** **4	1.07 (0.97–1.17)	1.07 (0.96–1.19)	1.46 (1.28–1.67)	1.18 (1.01–1.37)	1.38 (0.99–1.91)	2.36 (2.06–2.71)	1.69 (1.46–1.69)	1.71 (1.39–2.10)
** **5+	1.37 (1.11–1.69)	1.35 (1.07–1.71)	1.17 (0.82–1.66)	1.27 (0.88–1.84)	1.86 (0.91–3.79)	2.50 (1.86–3.35)	1.90 (1.37–2.62)	1.53 (0.94–2.50)
Continuous addition of each new medication class	1.03 (1.00–1.05)	1.02 (0.99–1.05)	1.10 (1.06–1.14)	1.06 (1.02–1.10)	1.09 (0.99–1.19)	1.32 (1.27–1.37)	1.19 (1.14–1.24)	1.21 (1.15–1.28)
Apparent treatment-resistant hypertension	1.10 (1.00–1.21)	1.10 (0.99–1.23)	1.42 (1.24–1.63)	1.21 (1.03–1.41)	1.49 (1.08–2.05)	2.30 (2.00–2.65)	1.76 (1.52–2.04)	1.79 (1.45–2.21)

Cardiovascular outcomes represents a composite of coronary heart disease, ischaemic stroke, and haemorrhagic stroke.

**Figure 2:**
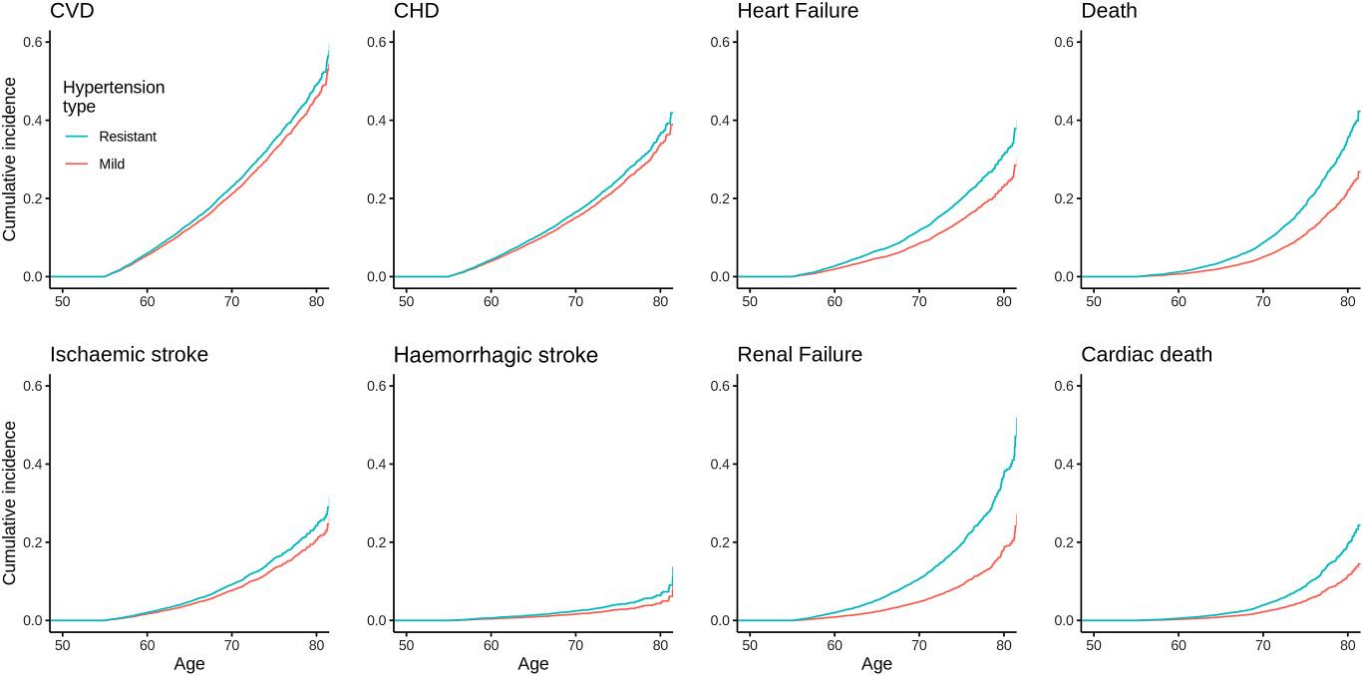
Cox proportional hazard models for adverse cardiovascular events over the lifespan, by hypertension subtype. Hard cardiovascular outcomes (CVD) represent a composite of coronary heart disease, ischaemic stroke, haemorrhagic stroke, death, and cardiac death. CVD, hard cardiovascular outcomes; CHD, coronary heart disease. Follow-up and risk of events begins at age 55 year for all participants.

Examination of risk among individuals with aRH demonstrated similar findings. Specifically, the lifetime risk of heart failure, renal failure, ischaemic stroke, haemorrhagic stroke, and CVD was all increased among aRH patients, with the largest risk appreciated for renal failure (2.3, 2.0–2.65), followed by cardiac death (1.79, 1.45–2.21), death (1.76, 1.52–2.04), intracranial haemorrhage (1.5, 1.08–2.05), and heart failure (1.4, 1.24–1.63) (*Table 3*; *Figure 2*, Supplementary material online, *Figure S2*).

### Sensitivity analyses

For renal failure, heart failure, death, and cardiac death results from all sensitivity analysis were similar to the main analysis (see Supplementary material online, *Tables S5–S7*), though both ischaemic and haemorrhagic stroke risks were no longer significant following the inclusion of socioeconomic variables into the model. Furthermore, when death was considered as a competing risk, or additional covariates added, the risks of CHD and CVD were not significant.

## Discussion

Our findings demonstrate that among an unselected cohort of adults with early onset hypertension, apparent treatment resistant hypertension is associated with increased cardiovascular risk across the lifespan. In particular, compared with hypertensive individuals receiving maximally one concurrent antihypertensive medication class prior to the age of 55, those with early onset aRH are at increased risk of suffering heart and renal failure, as well as ischaemic and haemorrhagic stroke, and both cardiac and all-cause death, with results robust to multivariable adjustment.

Prior short-term studies with selected patient samples have described increased cardiovascular risk associated with aRH. Among the largest of these, the Antihypertensive and Lipid-Lowering Treatment to Prevent Heart Attack Trial (ALLHAT) found a 44% increased risk of CHD, 88% increased risk of heart failure, and 95% increased risk of renal failure among apparent treatment resistant hypertensive patients with underlying kidney disease.^13^ Similar findings were appreciated among participants of the Chronic Renal Insufficiency and REGARDS Cohorts, among others.^14–16^ Importantly, follow-up of such studies is limited, with most providing far less than 10 years of follow-up data. Far greater is known regarding the lifetime risk of undifferentiated forms of hypertension (combined essential and resistant forms), with well-documented risk of myocardial infarction, heart failure, and stroke.^17–21^ Our study expands on this, first by examining a relatively young population, free from CVD. Such individuals represent a cohort that is yet to experience the sequela of long-term hypertension and may benefit from interventions to mitigate their risk. These include education on the importance of healthy lifestyle, medication adherence, and targeted evaluation for secondary forms of hypertension. Second, our results demonstrate that the elevated risk of adverse cardiovascular events associated with aRH persists overtime, above and beyond the cumulative risk associated with a lifetime of essential hypertension. In effect, those with essential hypertension do not ‘catch up’ to those with resistant hypertension with the risk of cardiovascular events remaining elevated for this population across the lifespan.

The greatest risk associated with aRH was appreciated for renal failure, with a hazard more than double that of those with hypertension on one medication class. This large increase in risk is notable and potentially reflective of the degree of renal parenchymal damage sustained from resistant disease. The association may also reflect the need for an increasing number of antihypertensive medications as renal disease progresses, representing a vicious cycle in which hypertension begets worsening renal function, which begets worsening hypertension. Importantly, hypertension remains one of the largest recognized risk factors for the development of renal failure and controlling blood pressure early in life represents one of the most cost-effective manners to prevent progression to end-stage kidney disease.^22–24^ As such, an early focus on aRH patients, particularly those under the age of 55, may help to reduce the burden of progressive kidney disease.

Hypertension also represents one of the largest risk factors for stroke, with attributable excess risk reaching as high as 76%.^25^ Prior work has identified a 14% increase risk of stroke among resistant hypertensive compared with non-resistant hypertensive patients over a 5-year follow-up period.^16^ Our results indicate that this risk may be even higher, reaching 21% for ischaemic stroke and nearly 50% for haemorrhagic stroke over the lifespan. Given the devastating effects of these events on longevity and quality of life, efforts at improving blood pressure control and minimizing other stroke risk factors is vitally important, particularly among those with apparent treatment resistant hypertension.

Interestingly, we did not appreciate a significant increase in CHD risk among those with aRH. As noted, resistant hypertension associated risk was lowest for the short-term development of CHD in the ALLHAT Study. Given the well-recognized association between hypertension and the development of coronary artery disease, a few factors may explain the lack of increased risk associated with resistant hypertension. First, CHD was the most common outcome of interest, reflective of its high prevalence in the population. Further, numerous other factors including family history, smoking, and lifestyle behaviours also contribute to the development of CHD, making detection of a resistant hypertension mediated association more difficult. Similarly, pluripotent effects of antihypertensive therapy may reduce CHD risk more than for other outcomes of interest. Finally, individuals with resistant hypertension may die from other conditions such as stroke or renal failure prior to the development of CHD, representing a survivor bias.

Several limitations of this study merit consideration. First, the administrative nature of the data necessitates reliance on ICD codes for the diagnosis of hypertension and outcomes of interest. Fortunately, coding in FinnGen has been found to be robust and reflective of true clinical events.^10^ Further, the prescription of antihypertensive medications also relies on administrative data, which do not include information on medication adherence or white coat effect. For this reason, we categorize our findings in the context of apparent treatment resistant and refractory hypertension, as delineated by current guidelines. While the FinnGen dataset represents an unselected cohort of the Finnish population, this group does not necessarily reflect the degree of racial and ethnic heterogeneity seen in other countries. Further, antihypertensive therapy guidelines contemporary to enrolment of most of our cohort participants prior to age 55 vary from current guidelines in regard to their recommendations for first line antihypertensive therapy.^26,27^ Specifically, beta blockers were highly recommended and combination therapy much less common. To harmonize our analysis with enrolment era guidelines, we included beta blockers for this reason. To counter the risk of non-hypertension reasons for drug prescription, we excluded individuals with cardiovascular disease at the time of enrolment, minimizing confounding by reason for prescription. Finally, we are unable to determine blood pressure control, comorbid disease severity, or antihypertensive medication adherence, limiting our ability to classify individuals as apparent treatment refractory of resistant hypertensives at lower medication class counts. To address this, we used conservative definitions, requiring higher medication class counts for each definition. Since blood pressure control, a key component of cardiovascular risk modification, cannot be verified, it is possible that those prescribed greater numbers of antihypertensive medications fail to achieve blood pressure targets to a greater extent than those on less complex regimens. As such, our results should serve to re-enforce the importance of appropriately tailoring antihypertensive therapies to achieve blood pressure control as a key factor in minimizing cardiovascular risk, particularly in early age.

In conclusion, the results from this large, unselected national dataset demonstrate that the risk of adverse cardiovascular events, particularly heart and renal failure, associated with aRH and refractory hypertension persists across the lifespan. Expanding on prior short-term studies, these findings demonstrate that even with lifetime exposure, hypertensive patients on fewer antihypertensive medications do not experience the amount of risk as those requiring more medications for hypertension management.

The association between aRH and risk of adverse cardiovascular events has been studied over short periods; however, little has been known about the associated risk across the lifespan. Our results indicate that compared to hypertensive individuals receiving maximally one concurrent antihypertensive medication, those with aRH remained at elevated risk of adverse events throughout their life course, with risk typically increasing with the addition of each subsequent medication. The greatest increase in risk was appreciated for renal failure, with those with aRH experiencing approximately twice the risk as those on only one antihypertensive medication. Importantly, this risk was appreciated even among a relatively young, healthy cohort, indicating that interventions aimed at controlling blood pressure, while minimizing the need for a greater number of antihypertensive agents early in life, may be beneficial. Whether this association reflects progressive end-organ dysfunction, particularly renal failure, necessitating more intense antihypertensive regimens, or individual differences in vascular biology with variable resistance to pharmacotherapy, requires future investigation. Clinicians should be mindful of patients suffering from aRH, focused not only of achieving blood pressure control, but to the use of non-pharmacologic interventions such as counselling on appropriate diet and exercise, to minimize the number of antihypertensive medications required to obtain that target.

## Supplementary material

Supplementary material is available at *European Journal of Preventive Cardiology* online.

## Supplementary Material

zwad066_Supplementary_Data

## Data Availability

Data for this study were obtained from the Finnish biobank, which can be accessed through the Fingenious® services (https://site.fingenious.fi/en/) managed by FINBB. A.K. and T.N. had full access to all the data in the study and take responsibility for the integrity of the data and the accuracy of the data analysis.

## Group author information

FinnGen: Aarno Palotie, Mark Daly, Bridget Riley-Gills, Howard Jacob, Dirk Paul, Athena Matakidou, Adam Platt, Heiko Runz, Sally John, George Okafo, Nathan Lawless, Robert Plenge, Joseph Maranville, Mark McCarthy, Julie Hunkapiller, Margaret G. Ehm, Kirsi Auro, Simonne Longerich, Caroline Fox, Anders Mälarstig, Katherine Klinger, Deepak Raipal, Eric Green, Robert Graham, Robert Yang, Chris O´Donnell, Tomi Mäkelä, Jaakko Kaprio, Petri Virolainen, Antti Hakanen, Terhi Kilpi, Markus Perola, Jukka Partanen, Anne Pitkäranta, Juhani Junttila, Raisa Serpi, Tarja Laitinen, Veli-Matti Kosma, Jari Laukkanen, Marco Hautalahti, Outi Tuovila, Raimo Pakkanen, Jeffrey Waring, Bridget Riley-Gillis, Fedik Rahimov, Ioanna Tachmazidou, Chia-Yen Chen, Heiko Runz, Zhihao Ding, Marc Jung, Shameek Biswas, Rion Pendergrass, Julie Hunkapiller, Margaret G. Ehm, David Pulford, Neha Raghavan, Adriana Huertas-Vazquez, Jae-Hoon Sul, Anders Mälarstig, Xinli Hu, Katherine Klinger, Robert Graham, Eric Green, Sahar Mozaffari, Dawn Waterworth, Nicole Renaud, Ma´en Obeidat, Samuli Ripatti, Johanna Schleutker, Markus Perola, Mikko Arvas, Olli Carpén, Reetta Hinttala, Johannes Kettunen, Arto Mannermaa, Katriina Aalto-Setälä, Mika Kähönen, Jari Laukkanen, Johanna Mäkelä, Reetta Kälviäinen, Valtteri Julkunen, Hilkka Soininen, Anne Remes, Mikko Hiltunen, Jukka Peltola, Minna Raivio, Pentti Tienari, Juha Rinne, Roosa Kallionpää, Juulia Partanen, Ali Abbasi, Adam Ziemann, Nizar Smaoui, Anne Lehtonen, Susan Eaton, Heiko Runz, Sanni Lahdenperä, Shameek Biswas, Julie Hunkapiller, Natalie Bowers, Edmond Teng, Rion Pendergrass, Fanli Xu, David Pulford, Kirsi Auro, Laura Addis, John Eicher, Qingqin S Li, Karen He, Ekaterina Khramtsova, Neha Raghavan, Martti Färkkilä, Jukka Koskela, Sampsa Pikkarainen, Airi Jussila, Katri Kaukinen, Timo Blomster, Mikko Kiviniemi, Markku Voutilainen, Mark Daly, Ali Abbasi, Jeffrey Waring, Nizar Smaoui, Fedik Rahimov, Anne Lehtonen, Tim Lu, Natalie Bowers, Rion Pendergrass, Linda McCarthy, Amy Hart, Meijian Guan, Jason Miller, Kirsi Kalpala, Melissa Miller, Xinli Hu, Kari Eklund, Antti Palomäki, Pia Isomäki, Laura Pirilä, Oili Kaipiainen-Seppänen, Johanna Huhtakangas, Nina Mars, Ali Abbasi, Jeffrey Waring, Fedik Rahimov, Apinya Lertratanakul, Nizar Smaoui, Anne Lehtonen, David Close, Marla Hochfeld, Natalie Bowers, Rion Pendergrass, Jorge Esparza Gordillo, Kirsi Auro, Dawn Waterworth, Fabiana Farias, Kirsi Kalpala, Nan Bing, Xinli Hu, Tarja Laitinen, Margit Pelkonen, Paula Kauppi, Hannu Kankaanranta, Terttu Harju, Riitta Lahesmaa, Nizar Smaoui, Alex Mackay, Glenda Lassi, Susan Eaton, Hubert Chen, Rion Pendergrass, Natalie Bowers, Joanna Betts, Kirsi Auro, Rajashree Mishra, Majd Mouded, Debby Ngo, Teemu Niiranen, Felix Vaura, Veikko Salomaa, Kaj Metsärinne, Jenni Aittokallio, Mika Kähönen, Jussi Hernesniemi, Daniel Gordin, Juha Sinisalo, Marja-Riitta Taskinen, Tiinamaija Tuomi, Timo Hiltunen, Jari Laukkanen, Amanda Elliott, Mary Pat Reeve, Sanni Ruotsalainen, Benjamin Challis, Dirk Paul, Julie Hunkapiller, Natalie Bowers, Rion Pendergrass, Audrey Chu, Kirsi Auro, Dermot Reilly, Mike Mendelson, Jaakko Parkkinen, Melissa Miller, Tuomo Meretoja, Heikki Joensuu, Olli Carpén, Johanna Mattson, Eveliina Salminen, Annika Auranen, Peeter Karihtala, Päivi Auvinen, Klaus Elenius, Johanna Schleutker, Esa Pitkänen, Nina Mars, Mark Daly, Relja Popovic, Jeffrey Waring, Bridget Riley-Gillis, Anne Lehtonen, Jennifer Schutzman, Julie Hunkapiller, Natalie Bowers, Rion Pendergrass, Diptee Kulkarni, Kirsi Auro, Alessandro Porello, Andrey Loboda, Heli Lehtonen, Stefan McDonough, Sauli Vuoti, Kai Kaarniranta, Joni A Turunen, Terhi Ollila, Hannu Uusitalo, Juha Karjalainen, Esa Pitkänen, Mengzhen Liu, Heiko Runz, Stephanie Loomis, Erich Strauss, Natalie Bowers, Hao Chen, Rion Pendergrass, Kaisa Tasanen, Laura Huilaja, Katariina Hannula-Jouppi, Teea Salmi, Sirkku Peltonen, Leena Koulu, Nizar Smaoui, Fedik Rahimov, Anne Lehtonen, David Choy, Rion Pendergrass, Dawn Waterworth, Kirsi Kalpala, Ying Wu, Pirkko Pussinen, Aino Salminen, Tuula Salo, David Rice, Pekka Nieminen, Ulla Palotie, Maria Siponen, Liisa Suominen, Päivi Mäntylä, Ulvi Gursoy, Vuokko Anttonen, Kirsi Sipilä, Rion Pendergrass, Hannele Laivuori, Venla Kurra, Laura Kotaniemi-Talonen, Oskari Heikinheimo, Ilkka Kalliala, Lauri Aaltonen, Varpu Jokimaa, Johannes Kettunen, Marja Vääräsmäki, Outi Uimari, Laure Morin-Papunen, Maarit Niinimäki, Terhi Piltonen, Katja Kivinen, Elisabeth Widen, Taru Tukiainen, Mary Pat Reeve, Mark Daly, Niko Välimäki, Eija Laakkonen, Jaakko Tyrmi, Heidi Silven, Eeva Sliz, Riikka Arffman, Susanna Savukoski, Triin Laisk, Natalia Pujol, Mengzhen Liu, Bridget Riley-Gillis, Rion Pendergrass, Janet Kumar, Kirsi Auro, Iiris Hovatta, Chia-Yen Chen, Erkki Isometsä, Kumar Veerapen, Hanna Ollila, Jaana Suvisaari, Thomas Damm Als, Antti Mäkitie, Argyro Bizaki-Vallaskangas, Sanna Toppila-Salmi, Tytti Willberg, Elmo Saarentaus, Antti Aarnisalo, Eveliina Salminen, Elisa Rahikkala, Johannes Kettunen, Mitja Kurki, Samuli Ripatti, Mark Daly, Juha Karjalainen, Aki Havulinna, Juha Mehtonen, Priit Palta, Shabbeer Hassan, Pietro Della Briotta Parolo, Wei Zhou, Mutaamba Maasha, Kumar Veerapen, Shabbeer Hassan, Susanna Lemmelä, Manuel Rivas, Mari E. Niemi, Aarno Palotie, Aoxing Liu, Arto Lehisto, Andrea Ganna, Vincent Llorens, Hannele Laivuori, Taru Tukiainen, Mary Pat Reeve, Henrike Heyne, Nina Mars, Joel Rämö, Elmo Saarentaus, Hanna Ollila, Rodos Rodosthenous, Satu Strausz, Tuula Palotie, Kimmo Palin, Javier Garcia-Tabuenca, Harri Siirtola, Tuomo Kiiskinen, Jiwoo Lee, Kristin Tsuo, Amanda Elliott, Kati Kristiansson, Mikko Arvas, Kati Hyvärinen, Jarmo Ritari, Olli Carpén, Johannes Kettunen, Katri Pylkäs, Eeva Sliz, Minna Karjalainen, Tuomo Mantere, Eeva Kangasniemi, Sami Heikkinen, Arto Mannermaa, Eija Laakkonen, Nina Pitkänen, Samuel Lessard, Clément Chatelain, Perttu Terho, Sirpa Soini, Jukka Partanen, Eero Punkka, Raisa Serpi, Sanna Siltanen, Veli-Matti Kosma, Teijo Kuopio, Anu Jalanko, Huei-Yi Shen, Risto Kajanne, Mervi Aavikko, Mitja Kurki, Juha Karjalainen, Pietro Della Briotta Parolo, Arto Lehisto, Juha Mehtonen, Wei Zhou, Masahiro Kanai, Mutaamba Maasha, Kumar Veerapen, Hannele Laivuori, Aki Havulinna, Susanna Lemmelä, Tuomo Kiiskinen, L. Elisa Lahtela, Mari Kaunisto, Elina Kilpeläinen, Timo P. Sipilä, Oluwaseun Alexander Dada, Awaisa Ghazal, Anastasia Shcherban, Rigbe Weldatsadik, Kati Donner, Timo P. Sipilä, Anu Loukola, Päivi Laiho, Tuuli Sistonen, Essi Kaiharju, Markku Laukkanen, Elina Järvensivu, Sini Lähteenmäki, Lotta Männikkö, Regis Wong, Minna Brunfeldt, Hannele Mattsson, Kati Kristiansson, Susanna Lemmelä, Sami Koskelainen, Tero Hiekkalinna, Teemu Paajanen, Priit Palta, Kalle Pärn, Mart Kals, Shuang Luo, Vishal Sinha, Tarja Laitinen, Mary Pat Reeve, Marianna Niemi, Kumar Veerapen, Harri Siirtola, Javier Gracia-Tabuenca, Mika Helminen, Tiina Luukkaala, Iida Vähätalo, Jyrki Pitkänen, Marco Hautalahti, Johanna Mäkelä, Sarah Smith, and Tom Southerington.
